# Minimum Connected Dominating Set Algorithms for Ad Hoc Sensor Networks

**DOI:** 10.3390/s19081919

**Published:** 2019-04-23

**Authors:** Xuemei Sun, Yongxin Yang, Maode Ma

**Affiliations:** 1 School of Computer Science and Technology, Tianjin Polytechnic University, Tianjin 300387, China; lilyyang0446@163.com; 2 School of Electrical and Electronic Engineering, Nanyang Technological University, Singapore 639798, Singapore; EMDMa@ntu.edu.sg

**Keywords:** ad hoc sensor networks, maximum independent set (MIS), minimum connected dominating set (MCDS), minimum spanning tree, Steiner tree

## Abstract

To achieve effective communication in ad hoc sensor networks, researchers have been working on finding a minimum connected dominating set (MCDS) as a virtual backbone network in practice. Presently, many approximate algorithms have been proposed to construct MCDS, the best among which is adopting the two-stage idea, that is, to construct a maximum independent set (MIS) firstly and then realize the connectivity through the Steiner tree construction algorithm. For the first stage, this paper proposes an improved collaborative coverage algorithm for solving maximum independent set (IC-MIS), which expands the selection of the dominating point from two-hop neighbor to three-hop neighbor. The coverage efficiency has been improved under the condition of complete coverage. For the second stage, this paper respectively proposes an improved Kruskal–Steiner tree construction algorithm (IK–ST) and a maximum leaf nodes Steiner tree construction algorithm (ML-ST), both of which can make the result closer to the optimal solution. Finally, the simulation results show that the algorithm proposed in this paper is a great improvement over the previous algorithm in optimizing the scale of the connected dominating set (CDS).

## 1. Introduction

An ad hoc sensor network is a kind of network with features of large scale, self-organization, and freedom from support from infrastructure [1]. At first, it was widely used in military applications. In recent years, it has been extensively applied in civil and commercial business [2,3]. Due to its large-scale and self-organized features, it can be automatically constructed in a very short time. However, its excessive ineffective transmission cannot be avoided, and a network storm may be caused easily. Meanwhile, due to its limited storage capacity and power resources of network nodes, it loses its transmission efficacy and the Internet link fails further. Eventually, the whole network may change. Therefore, it has become a favorable research method for researchers to construct a virtual backbone network [4,5,6] based on ad hoc sensor network. Connected backbone nodes in networks can perform efficient routing and broadcasting [7]. Virtual backbone networks can better adapt to changes of network topology and reduce communication costs of networking, which can reduce the inconvenience of resource utilization and network administration brought by its dynamic characteristics. In short, a virtual backbone network is the approximation of the network, which can be easily utilized to administer the entire network through the virtual backbone network. Research shows that the virtual backbone can play a vital role in the network routing of wireless ad hoc network routing, broadcasting, and connection control. In short, a virtual backbone network is an approximation of the network, which can be easily utilized to administer the entire network through the virtual backbone network.

In general, ad hoc sensor networks are composed of multiple nodes randomly distributed in a certain area, and they can communicate in two ways. If a pair of nodes are within the communication range, they can communicate directly; otherwise, they can communicate through the forwarding of intermediate nodes. The connected dominant set (CDS) becomes the best choice for an ad hoc sensor network as the virtual backbone network [8,9,10], which guarantees the operation of the network by constructing the minimum connected dominant set (MCDS).The purpose of this paper is to ensure the connection of communication network links through different topological structure control, so as to improve the network performance and effectively increase the coverage of the network. Therefore, the question of connectivity is critical.

Under the condition that all the requirements are met, the number of the connected dominating set nodes should be as small as possible so that more nodes can save energy by periodic dormancy. Meanwhile, it can also reduce the size of the routing table, reduce the cost of computation, and maintain the cost of routing tables. A minimum connected dominating set (MCDS) problem is a study that tries to seek a CDS which can meet such conditions and own the minimum number of nodes [11]. It is a NP hard problem to solve the connected dominating set [12]. Therefore, the researchers focus on how to solve the problem of approximate minimum connected dominant set. In the construction of virtual backbone network, in addition to considering the size of connected dominant set, but also combined with the actual situation as far as possible to balance the network energy consumption and load, extend the life of the network. Hence, researchers provide approximate solutions of this problem through different algorithms. The size of CDS (i.e., the number of nodes) serves as a standard to measure an algorithm. A smaller size of CDS means easier control of the network, since it can save energy by effectively reducing the unnecessary routing circulation. By analyzing the existing algorithms, it is concluded that the most effective method for solving the MCDS is based on the two-stage method, that is, an MCDS is obtained through the construction of a maximum independent set (MIS) in the first stage and the connection with the MIS by adding a Steiner node to construct a Steiner tree [13,14] in the second stage.

This paper proposes three improved algorithms for the two stages of the construction of MCDS. The main contributions are as follows:

(1) For the first stage, an improved collaborative coverage algorithm (IC-MIS) for solving the MIS is proposed. The algorithm uses a new dominating node selection scheme to choose the next dominating nodes from the three-hop neighbor of the current dominating nodes, and if it does not exist, the next dominating nodes will be chosen from the two-hop neighbor of the current dominating nodes. The overlap coverage between dominating nodes is reduced and the coverage efficiency is improved under the condition of complete coverage.
(2) For the second stage, an improved Kruskal–Steiner tree construction algorithm (IK–ST) is proposed. Most researchers generally use greedy method to construct and solve Steiner nodes, but this kind of algorithm is easy to fall into local optimal solution. In this paper, we improve the idea of IK–ST based on the spanning tree, add the corresponding weight strategy to the nodes selection, and realize the construction of the Steiner tree by adding the nodes replacement edge, In this way, the number of Steiner nodes will be further reduced.
(3) For the second stage, a new Steiner tree construction algorithm, a maximum leaf nodes Steiner tree construction algorithm (ML-ST), is also proposed, which is based on the idea of the maximum leaf nodes tree and sets the weight value for each edge according to a certain strategy. Then, a Steiner tree is generated by merging the edges with large weights to find the Steiner nodes, so that the results are closer to the optimal solution.
(4) Finally, the simulation experiments results show that the proposed algorithm is better than the typical collaborative coverage algorithm in optimizing the size of connected dominating set.

This paper is organized as follows: in Section 2, we discuss the relevant research on the CDS construction home and abroad. In Section 3, we introduce the details of network models and some symbol definitions. Section 4 introduces the concept and the steps of IC-MIS algorithm construction in details. In Section 5, the details of the IK–ST and ML-ST algorithms are presented. In Section 6, the experimental results are presented to prove that the algorithms we proposed are highly efficient. Finally, Section 7 concludes this paper.

## 2. Related Work

Various methods of CDS construction in literature can be classified into centralized algorithm and distributed algorithm according to the network information used. Among them, distributed algorithm is most widely used in CDS construction. Two centralized algorithms were first proposed by S. Guha in 1996 [15], aiming to construct CDS in a general undirected graph, and the performance of the algorithm was analyzed. However, the number of nodes in the dominating set obtained by this algorithm is relatively large, and the result is not very satisfying. The concept of ad hoc virtual backbone network [16] was firstly proposed by B. Das and V. Bharghavan. Different from the traditional concept of backbone network, an ad hoc virtual backbone network is not mainly used to exchange and forward data, but mainly responsible for the calculation and maintenance of the routing of ad hoc network. However, considering that the centralized algorithm requires the information of the entire global network, it is not suitable for ad hoc sensor networks. Based on the limitations of each algorithm, researchers [17] use the distributed method to build a centralized algorithm construct MCDS on the basis of research done by S. Guha and S. Khulle. Wu proposed a simple and realizable distributed CDS algorithm [18] in 2002. The algorithm first calculates the nodes of CDS, then deletes redundant nodes. The number of dominating set nodes obtained by this algorithm is relatively small, but there was still room to improve. In [19], L. Ruan proposed a new algorithm for solving MCDS by a stage, and the algorithm achieved good results. In 2001, I. Stojmenovic et al. proposed the idea of constructing CDS by two stages, that is: to realize the coverage of the whole network node by building a MIS first, then connect by adding additional nodes, and put forward the corresponding distributed MCDS algorithm [20]. The algorithm has achieved good results, so that the number of the obtained dominating set is further reduced. Then, the algorithm based on MIS is favored by researchers. In 2002, another two-stage distributed algorithm was proposed to construct the MCDS by Cardei et al. [21]. The algorithm uses the one-hop information of nodes to solve the MCDS problem, and the effect of the algorithm is further improved. After that, in [22], Li Y. et al. proposed a CDS algorithm based on MIS in 2005. The algorithm first constructs an MIS, and then realizes the connectivity of MIS nodes by an approximation algorithm, and experiments in his research show that the algorithm achieves good results. Among all the approximation algorithms for distributed CDS construction in UDGs, the most effective one is the one proposed by Rajiv Misra to construct the connected dominating set based on the heuristic algorithm of collaborative coverage. In [23], Rajiv Misra proposed a new heuristic algorithm for collaborative coverage. The algorithm uses the collaborative coverage method to obtain an MIS and realizes the connectivity of MIS by adding Steiner nodes. This algorithm is an improvement of the degree-based heuristic algorithm and the ID-based heuristic algorithm, and a smaller scale of CDS is obtained. Among all these algorithms, the most effective one is the one proposed by Rajiv Misra to construct the connected dominating set based on the heuristic algorithm of collaborative coverage. The algorithm mainly uses the theory of effective coverage to optimize the construction of MIS, but it does not optimize the Steiner nodes. Then, based on the research of Rajiv Misra, different algorithms are proposed in [24,25]. An improved ant colony algorithm is proposed in [24] to solve the MCDS issue, but this algorithm is based on the idea of constructing CDS by one stage. Using energy as a limiting factor, an energy-efficient method for constructing MCDS is proposed in [25], but the size of CDS is not further optimized. The authors of [26] propose a new degree-based greedy approximation algorithm named “Connected Pseudo Dominating Set Using 2 Hop Information”, which reduces the CDS size. In [27], a non-trivial potential function is proposed to increase the connectivity of a CDS. In this paper, based on the research of Rajiv Misra, three improved algorithms are proposed to construct MCDS.

## 3. Network Model, Dominating Set and Symbol Definitions

This section first introduces the ad hoc sensor network model, then presents related knowledge of dominating set, and finally provides the formalized definitions of the symbols mentioned in this paper.

### 3.1. Related Knowledge

**Figure 1:**
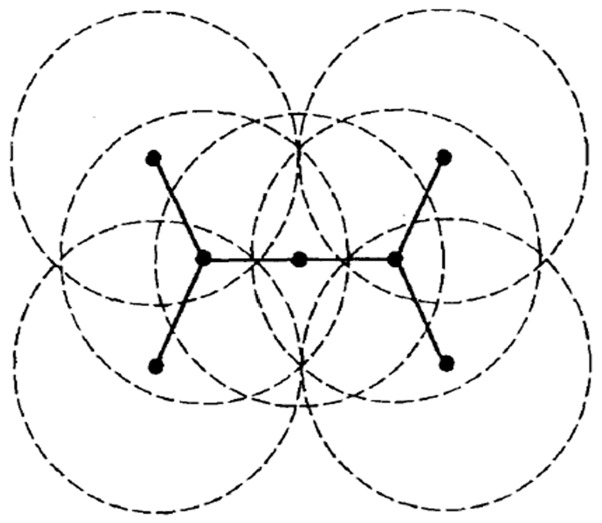
Unit-disk graph.

Assuming that all nodes in the ad hoc sensor network are distributed in a two-dimensional plane, each node has an omnidirectional antenna, the communication range of the node is defined as follows: take the node as the center, and the radius is equal to the transmission distance *R* of the node. The ad hoc sensor network can be abstracted into a unit-disk graph (UDG) when the transmission distance *R* of each node is fixed. In UDG graph, the topology of an ad hoc sensor network is represented by *G* = (*V*, *E*), where *V* denotes vertex set and *E* denotes edge set, and each node has a unique identifiable ID. For each pair of nodes *u*, *v* ∈ *V*, only if the distance between *u* and *v* is less than *R*, it is considered that there exists an edge in *u* and *v*, *e* = (*u*, *v*) ∈ *E*. Figure 1 is a simple UDG graph.

A dominating set for a graph *G* = (*V*, *E*) is a subset *V’* of *V* such that every vertex not in *V’* is adjacent to at least one member of *V’*, and then a spanning subgraph of graph *G*, *G’* = (*V’*, *E’*) is called a CDS. The maximum independent set MIS is also a dominating set, and it is required that any two nodes in the MIS do not have any adjacency, and if any node is added, there will be adjacency relation between two nodes in the MIS.

**Figure 2:**
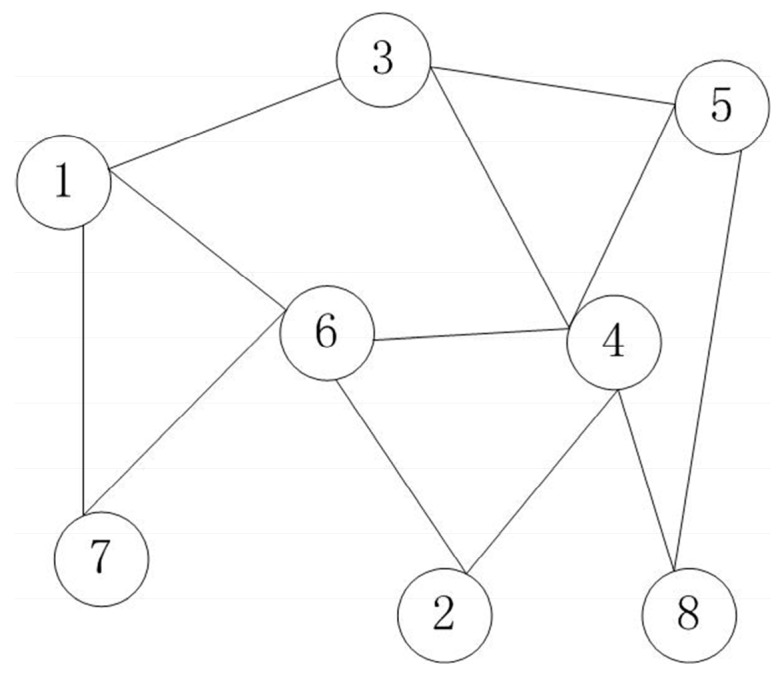
Dominating sets of graph *G*.

In Figure 2, {5,6}, {3,2,7}, and {6,4} are three dominating sets of graph *G*, where {5,6} is also called a MIS of graph *G*, {6,4} is called a CDS of graph *G*.

### 3.2. Formalized Definitions

In this paper, each different node type has a different color. The color state of the node represents the type of the node. There are five colors of nodes in this paper, white, black, blue, red, and gray. When initialized, all nodes states are white, and the dominating node’ s color state is black. For nodes that can receive broadcast messages within two-hop range of dominating nodes, its color state is blue. For nodes determined as the undetermined dominating node within three-hop range of the dominating node, their color state is red and the color state of dominated nodes is gray.

**Definition** **1.** *The graph G = (V, E) is a simple undirected graph, which needs to satisfy that the graph G is connected, and there can be at most one edge between any two nodes.*

**Definition** **2.** *If there is an edge between node u and node v in graph G, the node u and node v are adjacent.*

**Definition** **3.** *In graph G = (V, E), for any node v, the degree of node v is defined as the number of nodes adjacent to node v.*

**Definition** **4.** *In graph G = (V, E), if the color state of the neighbor node of node v is current_station, the Nv(current_station) represents the number of neighbor nodes whose color state are current_station within a hop range.*

**Definition** **5.** In graph G = (V, E), Wv represents the weight of node, Wv = 2*N_v(black)_ + N_v(gray)_, where N_v(black)_ represents the black neighbors of node v and N_v(gray)_ represents the gray neighbors of node v. This weight value is measured whether the node is suitable as a connected node.

**Definition** **6.** *We_1_ = 1/(Nu ∩ Nv), which represents the weight of the edge e = (u, v) of the spanning tree. In the IK–ST algorithm, it is used as the value of the edge in constructing the spanning tree.*

**Definition** **7.** *We_2_ = Wv + Wu, which is used to define the merging weight value of the edge e = (u, v). In IM-ST algorithm, it is used as the measure of merging edge.*

**Definition** **8.** *Pro(v) represents the priority weight of node v, Pro(v) = N_v(white)_ + N_v(blue)_, where N_v(white)_ and N_v(blue)_ respectively represent number of white neighbors and blue neighbors of node v.*

## 4. IC-MIS Algorithm

**Figure 3:**
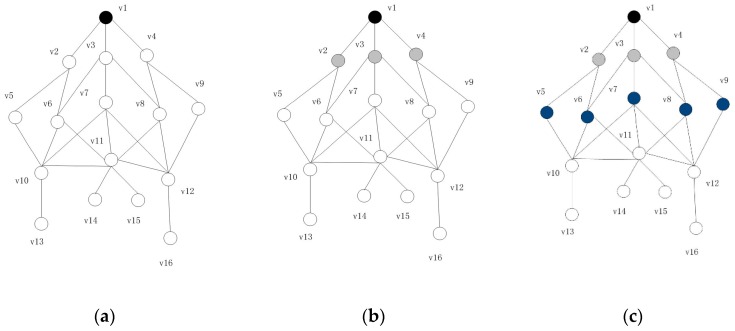

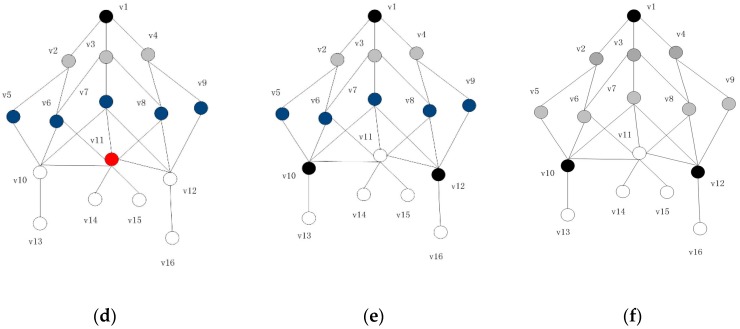
Examples of IC-MIS algorithm: (**a**) Initially, all nodes are white, and *v_1_* as the first dominating node, set state as black; (**b**) *v_1_* broadcasts message, *v_2_*,*v_3_*,*v_4_* receive the message, set state as gray; (**c**) *v_2_*,*v_3_*,*v_4_* broadcast messages, *v_5_*,*v_6_*,*v_7_*,*v_8_*,*v_9_* receive the message, set state as blue; (**d**) *v_11_* has the high *Pro*, set state as red; (**e**) *v_10_* and *v_12_* as the next real dominating nodes; (**f**) when *v_10_* and *v_12_* as the dominating nodes, update *v_5_*,*v_6_*,*v_7_*,*v_8_* as gray.

Presently, many papers have put forward algorithms to solve CDS based on MIS, among which the collaborative coverage heuristic algorithm proposed by Rajiv Misra has the best effect in constructing MIS. The algorithm takes into account that when nodes are considered separately, the coverage efficiency is low. At the same time, for a connected graph *G*, the number of slopes is greater than or equal to 2, that is, *G* has at least two coverages. Therefore, the algorithm optimizes the whole MIS by finding the independent set of neighbor nodes. However, the algorithm selects the next dominating node from the two-hop neighbor of the current dominant nodes, which results in the intersection of the dominating node coverage and reduces the coverage efficiency. The IC-MIS algorithm preferentially selects the next dominating node from the three-hop neighbor of the current dominating node, and if it does not exist, it selects the next dominating node from the two-hop neighbor of the current dominating node, so that it greatly increases the efficiency of coverage for there is as little coverage intersection as possible. Figure 3 is an example of the IC-MIS algorithm.

**Algorithm 1:** IC-MIS algorithm1:**Input**: graph *G* = (*V*, *E*), initialize set *D* is empty, state of all nodes is set to white.2:Starting from the base station node *B*, node *B* is added to the set *D* as the first dominating node. Node *B* update its own state to black, and broadcast the message *m_1_* to the neighbor node in the one-hop range;3:After receiving *m_1_*, each adjacent node sets its state to gray and broadcasts the message *m_2_* to the surrounding hop neighbor nodes, each white node sets its state to blue after receiving *m_2_* message;4:Each blue node continues to broadcast message *m_3_* to nearby one-hop. After receiving the m_3_ message, node *t* calculates priority *Pro*(*t*) according to Definition 8. All blue nodes elect a white node *Dt* with the highest priority through comparison *Pro*(*t*) as an undetermined dominating node, and the status of the node is set to red. If the priority is equal, the node with a larger degree will be chosen; if the node degree is equal, the node with small ID is given priority. If there is no node *D_t_* under white status, the node with the highest *Pro*(*v*) from blue nodes is chosen as *D_t_*;5:When the *D_t_* node is selected, we calculate the independent set IS of the *D_t_* node in one-hop range through the message broadcast; there will be many IS at this time, and we choose the largest *W_IS_* (*W_IS_* = the number of nodes covered / the number of dominating nodes) to replace the *D_t_* and restore the state of the *D_t_* node to white, where the nodes in IS are chosen as the dominating nodes. Set the state of the node in IS as black and update the node in one-hop as gray;6:Repeat the above steps until no white node exists in all nodes.

**Figure 4:**
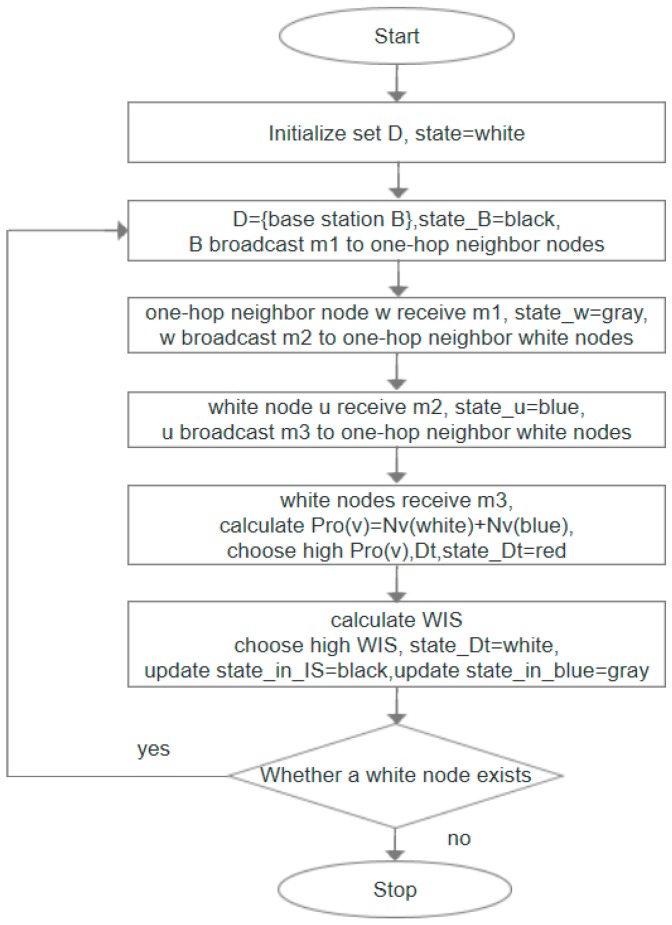
Flowchart of IC-MIS algorithm. The node that receives the broadcast message continues to broadcast the message to its one-hop neighbors.

Figure 4 is the flow chart of IC-MIS algorithm.

Initially, all nodes are white, starting with base station node *v_1_* as the first dominating node, setting the state as black, such as Figure 3a; *v_1_* broadcasts messages outwards, the node that receives the broadcast messages sets the state to gray, such as *v_2_*,*v*_3_,*v*_4_ in Figure 3b; all gray nodes continue to broadcast messages, the white nodes that receive the messages are set to blue, such as *v_5_*, *v_6_*, *v_7_*, *v_8_*, *v_9_* in Figure 3c; when the blue node broadcasts the message outwards, the white nodes that receive the message quickly reply to the blue node, the blue node collects the message and calculates the priority *Pro* of the nodes, notifies the highest node of *Pro*, *v_11_*, as the undetermined dominating node, sets the state to red, and broadcasts the message out, such as Figure 3d; the white node that receives the undetermined message broadcasts the message outward and calculates the *W_IS_* (*W_IS_* = the number of nodes covered / the number of dominating nodes) value of its own replacement node, such as Figure 3e, *v_10_* and *v_12_* are selected to join the IS set as the next real dominating node.

So far, the two-hop nodes based on the base station have been completely covered and the final MIS includes *v_1_*, *v_10_*, *v_12_*. The newly-selected dominating nodes *v_10_*, *v_12_* carry out the next broadcast. In this way, the coverage efficiency is greatly improved by selecting the next dominating node from within three hops. At the same time, by replacing the undetermined dominating node with IS, getting into the local optimum is avoided and the final result is further optimized. Then the detailed steps of the algorithm are introduced in Algorithm 1.

## 5. Steiner Tree Construction Algorithm Based on Minimum Spanning Tree

**Figure 5:**
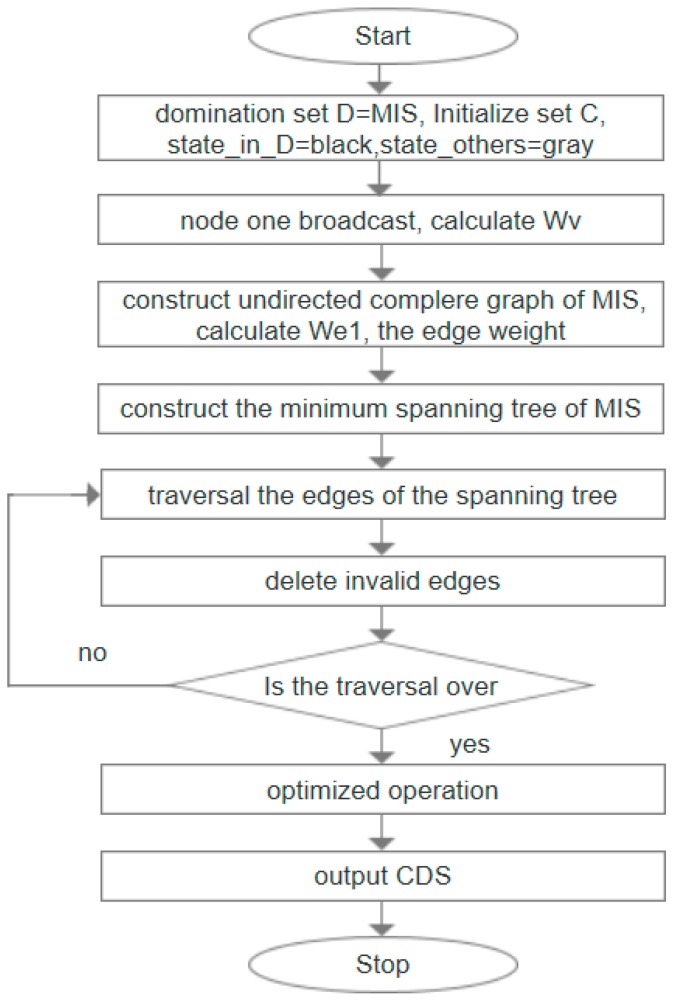
Flowchart of IK–ST algorithm.

**Figure 6:**
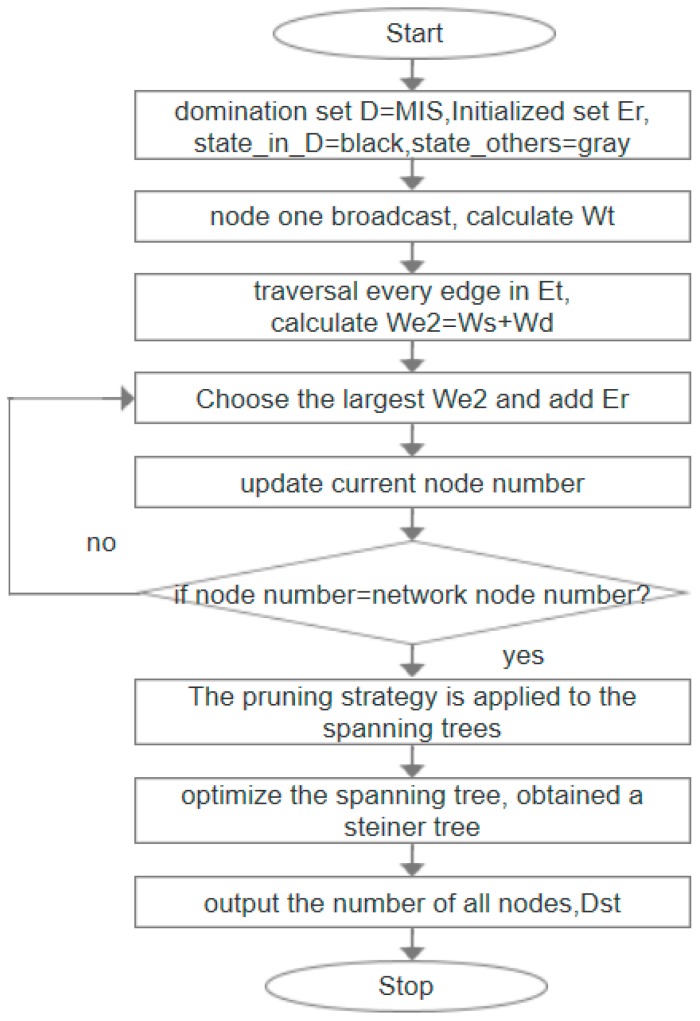
Flowchart of ML-ST algorithm.

The algorithm of Steiner tree construction aims to connect the obtained MIS by adding Steiner nodes to construct a Steiner tree, finally, the set of all nodes obtained by the Steiner tree is CDS. In this section, two algorithms, IK–ST and ML-ST are proposed, and the following two algorithms are introduced in detail. Figure 5 and Figure 6 are the flow chart of IK–ST algorithm and ML-ST algorithm, respectively.

### 5.1. IK–ST Algorithm

In the second phase of the Rajiv Misra algorithm, Rajiv Misra adds extra nodes by using the approximate greedy method to realize the connection, which is easy to get into the local optimum and lead to the existence of redundant nodes. IK–ST uses the idea of minimum spanning tree to solve Steiner nodes and realize the connection of MIS. It is believed that a gray node is more suitable to be a connecting node, which can be adjacent to more black nodes. The algorithm first sets the weights of MIS nodes and then constructs an undirected graph of MIS, setting a weight value for each side. We utilize the classical Kruskal algorithm to construct a minimum spanning tree, and search nodes from any edge to replace the edge of the spanning tree, finally, we optimize the node.

The details of the IK–ST algorithm are summarized in Algorithm 2:

**Algorithm 2:** IK–ST algorithm1:**Input**: graph *G* = (*V*, *E*), dominating set *D* = MIS; initialize one set *C* as the node empty; nodes in *D* are black, and the rest of the nodes are gray.2:First of all, set the weight *Wv* for each node. The *Wv* can be set by one broadcast of the node, and the rules are calculated according to Definition 5;3:The undirected complete graph of MIS is constructed, and the weight value *W_e1_*(*s*, *d*) of each edge is calculated according to Definition 6 (if node *s* and node *d* do not have a common join node, set the weight *W_e1_* of this edge to a maximum);4:The construction method adopts the classical Kruskal algorithm to construct the minimum spanning tree of MIS, T_MIS_;5:Select any edge *E* from the edge set of the spanning tree, where *s* and *d* are the source node and destination node of the edge. Select a node *t* from one-hop neighbor of *s*, and make sure that *s* can be accessed to *d* through two hops. The selection rule is as follows: select the node that can connect the biggest number of black nodes except *d* and *s* nodes, if equal, the node with a small node ID is preferred;6:When the *t* node is found, *t* is added to *C* and then delete the invalid edge. The strategy employed is as follows: firstly, delete the current edge; secondly, if *t* has an adjacent node except *s* or *d*, and the node has an edge *E’* or the node is adjacent to *s* or *d*, then we delete *E’*;7:Repeat 4 and 5 until all the edges of the spanning tree are traversed;8:In the optimization stage, the nodes in *C* are traversed in turn. If all the black nodes adjacent to a node *t’* can be adjacent to the nodes in *C*\{t}and *C*\{t}can still form a spanning tree, then delete *t’*.9:**Output**: *D* + *C* i.e., (CDS)

### 5.2. ML-ST Algorithm

In a given graph *G*, suppose there is a spanning tree *T*, the sum of the degree of the spanning tree *T* is equal to the number of leaf nodes plus the number of non-leaf nodes multiplied by the degree of non-leaf nodes, if the number of leaf nodes is required to be as large as possible, the number of non-leaf nodes will be reduced and the degree of non-leaf nodes should be as large as possible.

According to this idea, considering the CDS problem is one to be solved in this paper, if the whole network is regarded as an undirected graph, then the virtual backbone network formed by this CDS is equivalent to a spanning tree of graph *G*, the dominating node is equivalent to the non-leaf node in the tree, and the dominated node is equivalent to the leaf node in the tree. Therefore, the problem of solving the CDS problem can be transformed into the problem of solving a spanning tree with the most leaf nodes.

The details of the ML-ST algorithm are summarized in Algorithm 3:

**Algorithm 3** ML-ST algorithm1:**Input**: graph *G* = (*V*, *E*), dominating set *D* = MIS; initializing a set *Er* to be empty, setting the state of nodes in *D* as black and the rest as gray.2:Initialize an edge set *E_t_* and add all existing edges in the graph to *E_t_* by a broadcast of the node;3:The weight value *W_t_* of each node is calculated by a broadcast message of each node, and the standard of calculation is Definition 5, which ensures that the edges adjacent to the midnode of MIS can be selected into the spanning tree first;4:Traverse each edge in *Et* and set weight values for each edge as the standard of merging the spanning tree for the next step, we select a side *e*, *W_e2_* = *W_s_* + *W_d_* by the calculation of the edge weight according to Definition 7, where *W_s_* and *W_d_* are the weights obtained in Step 2, while *W_s_* represents the weight of the source nodes of *e*, and *W_d_* represents the weight of the destination nodes of *e*;5:Start from any node in MIS and traversing the edge of *Et*, the edge with maximum *W_e2_* is selected to be added to *Er* for each time, and if equal, any one edge is added. Update the current number of nodes until the number of nodes is equal to the number of network nodes;6:A pruning strategy is made for the spanning tree formed by edges in *Et*. The pruning strategy is as follows: traversing each node, if a node has a degree of 1 and the node is not a black node, he node and its associated edges are deleted;7:Optimize the spanning tree after processing of Step 5 and traverse the nodes in the spanning tree in turn. If all the black nodes adjacent to one node *t’* can be adjacent to the other nodes, and can still form a spanning tree, then delete *t’*. It is obtained that the last *E_r_* edge set constitutes a Steiner tree, in which the number of all nodes are recorded as *D_ST_*.8:**Output**: *D_ST_*

## 6. Simulation Experiments

In this section, three experiments were conducted to verify the feasibility and validity of our algorithm. In each experiment, the collaborative coverage algorithm proposed by Rajiv Misra is chosen to make the comparison. The first experiment mainly evaluates the algorithms through comparing the number of sensor nodes for constructing MIS. In the second experiment, we use IK–ST and ML-ST algorithms separately to optimize the Steiner tree construction phase of Rajiv Misra algorithm. The second experiment mainly evaluates the efficiency of the proposed algorithms. In the third experiment, we combine the IC-MIS algorithm with IK–ST and ML-ST algorithm respectively and use the IC-MIS algorithm to calculate the value in the first stage, and we use the IK–ST algorithm and ML-ST algorithm to calculate the value in the second stage of Steiner nodes respectively. The algorithms are evaluated according to the number of nodes for constructing CDS. The simulation experiment mainly compares two aspects: (1) the number of nodes for constructing MIS in the first stage of solving CDS; (2) the number of Steiner nodes obtained in the second stage of solving CDS.

In this paper, the ad hoc network is simulated as a 100 × 100 rectangular area with a random deployment of sensor nodes, and the whole network is guaranteed to be a connected graph. We assume that the communication radius of each sensor node is a constant value *r*, if the distance between the sensor node *u* and sensor node *v* is less than *r*, it is considered that there is an edge between *u* and *v*, so the whole network is abstracted into a unit disk graph. In the experiment, the communication radius of the sensor node is set to 25, 30, 35, or 40. The number of network nodes is set to 25, 50, 100, or 150 respectively. The parameters of all experiments were listed in Table 1. Then the algorithm runs 1000 times respectively, and the average value of the experiment is obtained. The details of the experiment are as follows.

**Figure 7:**
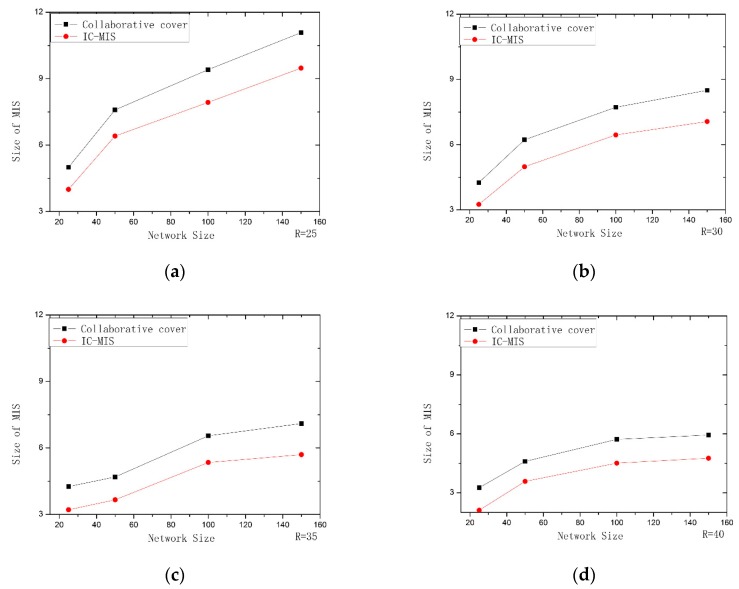
Performance comparison of IC-MIS with Collaborative coverage: (**a**) R = 25; (**b**) R = 30; (**c**) R = 35; (**d**) R = 40.

In the first experiment, we compared the IC-MIS algorithm and the algorithm proposed by Rajiv Misra for constructing MIS. This experiment mainly evaluates the algorithms through comparing the number of sensor nodes for constructing MIS. The results of simulation experiment are shown in Figure 7. We can find the following results from these figures: (1) When the communication radius of sensor node is a fixed value, the number of sensor nodes required for constructing MIS by these two algorithms increases with the increase of the number of sensor nodes, furthermore, the number of nodes required for constructing MIS by IC-MIS increases significantly less; (2) When the number of sensors deployed in the network is the same, with the communication radius of sensors in the network increases, the number of nodes required for the construction of MIS by these two algorithms is reduced, and the number of nodes required for constructing MIS by IC-MIS is significantly reduced. The reason for this is that IC-MIS algorithm reduces overlapped coverage as far as possible, the number of MIS decreases relatively, and the coverage efficiency improves. The effect of IC-MIS algorithm is significantly superior to Rajiv Misra’s algorithm for constructing MIS. The IC-MIS algorithm can acquire smaller-scale CDS.

**Figure 8:**
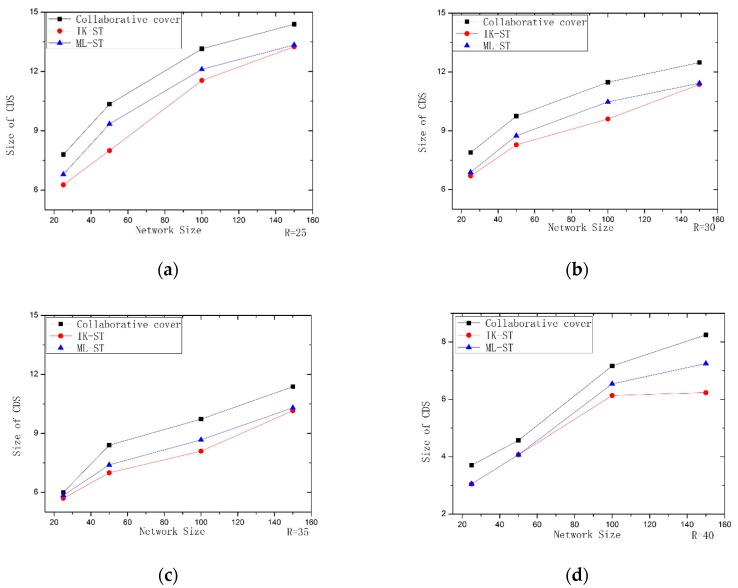
Performance comparison of IK–ST, ML-ST with Collaborative coverage: (**a**) R = 25; (**b**) R = 30; (**c**) R = 35; (**d**) R = 40.

In the second experiment, we use separately IK–ST and ML-ST algorithms to optimize the Steiner tree construction phase of Rajiv Misra algorithm, and then compare them with it and evaluate the efficiency of the proposed algorithms. In the experiment, the size of the CDS (the number of nodes in CDS is equal to the number of nodes in MIS plus the number of nodes in Steiner tree, where the number of nodes in MIS is the same) is used to evaluate the performance of the algorithm. The simulation results are shown in Figure 8. It can be seen from the figures that when the number of network nodes is small, the number of connected nodes obtained by the three groups is not much different. However, with the increase of the number of nodes, the effects of IK–ST algorithm and ML-ST algorithm are obviously better than that of Rajiv Misra algorithm. It shows that IK–ST and ML-ST can avoid falling into a local optimal solution and realize the optimization of node number, in which IK–ST has the best effect. The number of CDS can be obtained for a smaller number of nodes by IK–ST algorithm.

**Figure 9:**
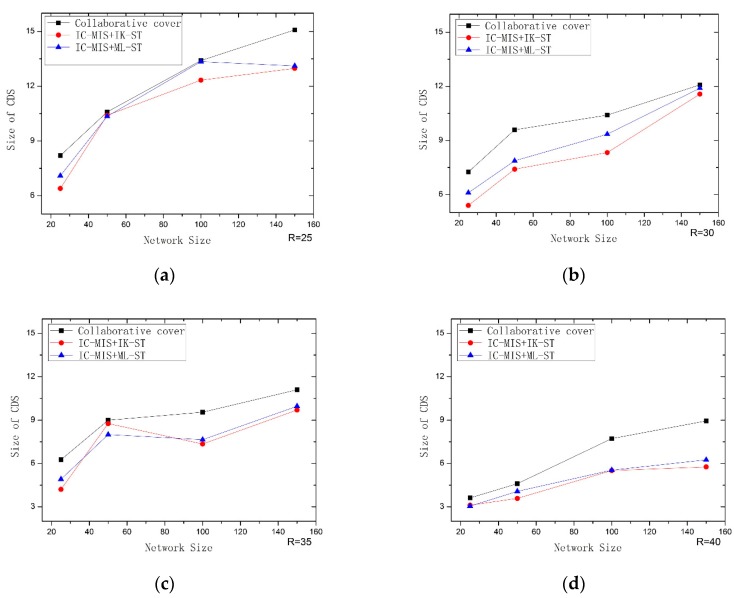
Performance comparison of IC-MIS+IK–ST, IC-MIS+ML-ST with Collaborative coverage: (**a**) R = 25; (**b**) R = 30; (**c**) R = 35; (**d**) R = 40.

In the third experiment, we combine IC-MIS algorithm with IK–ST and ML-ST algorithm respectively and use the IC-MIS algorithm to calculate the value in the first stage, and we use the IK–ST algorithm and ML-ST algorithm to calculate the value in the second stage of Steiner nodes respectively. Then, we compared it with Rajiv Misra’s algorithm. The algorithms are evaluated according to the number of nodes for constructing CDS. The simulation results are shown in Figure 9. It can be seen from the figures that the combination of IC-MIS algorithm with IK–ST algorithm and ML-ST algorithm respectively have achieved better results, and with the increase of transmission radius, the difference of effect is more obvious. At the same time, when the transmission radius is constant, the effects of the two algorithms are better than that of Rajiv Misra’s algorithm with the increase of the number of nodes. The combination of the IC-MIS algorithm and IK–ST is correspondingly the best, that is, the number of nodes for constructing CDS by this algorithm is the least.

## 7. Conclusions

In the present study, three algorithms are proposed to reduce the number of CDS nodes in ad hoc sensor networks. Aiming at the goal of reducing the number of CDS nodes in ad hoc sensor networks, this paper improves and optimizes the number of CDS nodes from the two stages of constructing CDS. The first stage is to solve the MIS. In this paper, the existing minimum connected dominating set algorithm based on collaborative coverage is analyzed, we consider that when the nodes are considered in isolation, there would be less effective coverage. Thus, the important information of further optimizing the size of the maximum independent set and the connected dominating set would be lost. However, although the overlapping coverage idea of collaborative coverage algorithm can bring the problem close to the optimal solution, it also reduces the coverage efficiency at the same time. In order to solve this problem, a new scheme is adopted in this paper, which gives priority to selecting the next dominating node from the three-hop neighbor of the current dominating node. If it does not exist, the next dominating node is selected from the two-hop neighbor of the current dominating node. In this way, the coverage efficiency can be increased. The second stage is to build the Steiner tree. According to previous studies, greedy algorithm can be used to find nodes that can connect more dominating nodes as Steiner nodes. The advantage of greedy algorithm lies in its simple implementation, but it is easy to get into the local optimal solution, and at the same time, redundant connected nodes may be obtained. To solve this problem, this paper respectively proposes two improved Steiner tree construction algorithms—IK–ST algorithm and ML-ST algorithm. In our experiments, we compare the proposed algorithm with the collaborative coverage algorithm. In the first experiment, we find that the IC-MIS algorithm reduces overlapped coverage as far as possible, and the number of MIS decreases relatively, and coverage efficiency improves. Meanwhile, for the second experiment, the result shows that IK–ST and ML-ST can avoid falling into the local optimal solution and realize the optimization of node numbers, in which IK–ST have the best effect. In the last experiment, we combine the IC-MIS algorithm with the IK–ST algorithm and ML-ST algorithm respectively. As is shown in the result, the proposed algorithms achieve better results, and with the increase of transmission radius, the difference of effect is more obvious. According to these three experimental results, we can understand that the proposed algorithms can achieve a smaller scale of CDS.

## Figures and Tables

**Table 1:** Simulation parameters

Parameter	Value	Parametric Description
M	100 × 100	Rectangular network deployment area
r	25–50	Communication radius of each sensor node
n	25–500	Network size
d	3–50	Network density, Number of nodes/The unit area
